# The Institutional Worker

**Published:** 1912-09-28

**Authors:** 


### THE INSTITUTIONAL WORKER

**Being a Special Supplement to "The Hospital"**

### Editor's Notices.

**Contributions:**

Contributions should be written, or preferably typed, on one side of the paper only, and all articles sent in are accepted upon the distinct understanding that they are forwarded to The Hospital only.

The Editor cannot undertake to return MSS. not used, but when a stamped directed envelope is enclosed the MSS. may be returned if a special request is made.

Accepted articles and paragraphs of news will be paid for after publication at the scale rate.

**Address:**

To prevent delay all contributions and letters on editorial business must be addressed exclusively to the Editor, "The Hospital," 29 Southampton Street, Strand, London, W.C. It is important that this regulation shall be strictly observed.

**Correspondence:**

Correspondence on all subjects is invited. The name and address of correspondents must be given as a guarantee of good faith, but not necessarily for publication.

**Special Articles:**

Special articles are invited, and questions, inquiries, and paragraphs upon all matters relating to the work, administration, and management of general, special, mental, fever, cottage and convalescent hospitals, sanatoria, homes, institutions, societies and organisations for the treatment or care of the sick, injured and dependants of all classes. Every matter affecting the interests and welfare of the staff of all grades working in these institutions will receive special consideration.

**Administrative Medicine, Research and Items of News:**

Special terms will be paid for approved articles on subjects in this wide field by experts who have made some section of it their special study and interest. We wish to give prominence to every movement tending to advance accuracy of diagnosis, efficiency in treatment, and the development of modern methods to eradicate disease and promote the welfare of the suffering.

**A Bureau of Information:**

We invite inquiries and applications for information and help in providing for that numerous class of sufferers who require special care or house-room which they cannot obtain in their own homes or secure for themselves. **We cannot, however, prescribe, or recommend practitioners.**

**Books for Review:**

Publishers are particularly requested to send advance proofs of any new books of importance whenever possible, as the Editor has made arrangements to publish immediate reviews on a new plan.

**Photographs, Plans, Blocks, and Illustrations:**

It is requested that wherever possible MSS. may be accompanied by illustrations in any of the above forms. The name of the sender and of the article to which it belongs should be written on the back of each photograph, drawing, or block for purposes of identification.

**Local Papers:**

Newspapers containing reports or news paragraphs **should be marked** and addressed to the Sub-Editor.

**The Coupon System:**

A coupon will be found at the bottom of the third inside page of the cover each week. This coupon must be attached to every question or inquiry to which an answer is desired in The Hospital.

### The Institution and the Workers.

The success of *The Institutional Worker* as a means of announcing vacancies in every department of Institutional work is evidenced by the increasing number of advertisements that appear from week to week in this section of The Hospital. No more adequate proof of the value of *The Institutional Worker* can be desired than the fact that Authorities of the more important Institutions throughout the United Kingdom employ its columns again and again whenever they have a vacancy to fill upon their staffs. It also provides exceptional facilities for every class of announcement affecting Institutional Life and Work, and as a medium for securing appointments in any section of the Institutional, Health, and Sanitary Services it is absolutely incomparable, since no other journal possesses such a comprehensive circulation in these directions as The Hospital. If you are seeking a post an announcement of twenty-one words, costing the small sum of 1s., will save you many hours of anxious thought, and certainly bring your requirements immediately to the notice of those most likely to need your services.

The form below may be used for Appointments Wanted advertisements, and when filled up should be posted to the

*Manager*, The Hospital,
28 and 29 Southampton Street,
Strand, W.C.

Appointments Wanted, 21 words ... 1s. 0d.

| | | |
|---|---|---|
| | | |
| | | |
| | | |
| | | |
| | | |
| | | |
| | | |

### Manager's Notices.

**All letters on business matters must be addressed to The Manager, "The Hospital," 28 and 29 Southampton Street, London, W.C.**

**Advertisements:**

To ensure insertion, all advertisements for The Hospital must reach the Manager not later than Wednesday at noon in each week. For scale of charges see pages v and 2.

**Subscriptions**, which may commence at any time, are payable in advance. Cheques and Post-Office Orders should be crossed London County and Westminster Bank, Covent Garden Branch, and made payable to the Manager of The Hospital.

Rates of Subscription (including Postage).

| *United Kingdom.* | | *Foreign and Colonial.* | |
|---|---|---|---|
| Three Months ... ... | 2s. 0d. | Three Months ... ... | 2s. 6d. |
| Six Months ... ... | 4s. 0d. | Six Months ... ... | 5s. 0d. |
| Twelve Months ... ... | 6s. 6d. | Twelve Months ... ... | 8s. 8d. |

**Remittances:**

**It is especially requested that remittances be made by Cheque or Postal Order, and in halfpenny stamps only when the amount is under sixpence.**

### The Scientific Press Publications

### LEDGERS, ACCOUNT BOOKS, STOCK BOOKS of all kinds for large and small Institutions.

These are supplied already ruled and printed, or in accordance with the special requirements of institution managers.

### TEMPERATURE CHARTS.

MORNING, EVENING, FOUR-HOUR, MATERNITY.

Size 10 inches by 8 inches

| | | | | | |
|---|---|---|---|---|---|
| 12 | 3d. | 50 | 9d. | 250 | 3/3 |
| 25 | 6d. | 100 | 1/4 | 500 | 6/6 |
| | | | | 1,000 | 12/6 |

Postage extra.

All Orders for 250 and over are sent Carriage Paid.

### CASE PAPERS

For Day and Night Nurse's Reports. Bound & unbound.

### DISTRICT NURSING ASSOCIATIONS'

Case, Visit, Time, and Loan and Account Books.

### *INSTITUTIONAL LIBRARY.*

THE SCIENTIFIC PRESS will procure literature, professional and otherwise, for the formation of and addition to Institutional Libraries. It is economy of time and expenditure to utilise one source of supply.

**THE SCIENTIFIC PRESS, LTD.,**
**28 and 29 Southampton Street, Strand, London, W.C.**

Send for complete Catalogue.

***IMPORTANT TO HOSPITAL SECRETARIES.***

### ANALYSIS LEDGER.

Specially Ruled in accordance with, and adapted to the requirements of, the New Index of Classification recently adopted by the King Edward's Hospital Fund for London, and which is now in operation.

The Ledger is bound in half basil, green cloth sides, printed and ruled in best style on good paper, and is in every way a serviceable book for Hospital use.

**Price £1 12s. 6d.** *Carriage extra.*

Secretaries requiring a copy of this Ledger should order at once to avoid delay.

**THE SCIENTIFIC PRESS, LTD., 28 & 29 Southampton St., Strand, London, W.C.**

